# Involvement of *Arabidopsis thaliana* endoplasmic reticulum KDEL-tailed cysteine endopeptidase 1 (AtCEP1) in powdery mildew-induced and AtCPR5-controlled cell death

**DOI:** 10.1371/journal.pone.0183870

**Published:** 2017-08-28

**Authors:** Timo Höwing, Marcel Dann, Caroline Hoefle, Ralph Hückelhoven, Christine Gietl

**Affiliations:** 1 Technische Universität München, Center of Life and Food Sciences Weihenstephan, Lehrstuhl fürBotanik, Freising, Germany; 2 Technische Universität München, Center of Life and Food Sciences Weihenstephan, Lehrstuhl für Phytopathologie, Freising, Germany; University of Maryland Baltimore County, UNITED STATES

## Abstract

Programmed cell death (PCD) is a prerequisite for successful development and it limits the spread of biotrophic pathogens in a rapid hypersensitive response at the site of infection. KDEL-tailed cysteine endopeptidases (KDEL CysEP) are a subgroup of papain-type cysteine endopeptidases expressed in tissues undergoing PCD. In *Arabidopsis*, three KDEL CysEPs (*AtCEP1*, *AtCEP2*, and *AtCEP3*) are expressed. We have previously shown that AtCEP1 is a factor of basal resistance to powdery mildew caused by the biotrophic ascomycete *Erysiphe cruciferarum*, and is expressed in spatiotemporal association with the late fungal development on *Arabidopsis* leaves. The endoplasmic reticulum-localized proenzyme of AtCEP1 was further visualized at the haustorial complex encased with callose. The *AtCPR5* gene (*CONSTITUTIVE EXPRESSION OF PR GENES 5*) is a regulator of expression of pathogenesis related genes. Loss of *AtCPR5* leads to spontaneous expression of chlorotic lesions which was associated with enhanced expression of *AtCEP1*. We used the *atcpr5-2* mutant plants and the *atcep1 atcpr5-2* double mutants harboring a non-functional reporter (P_CEP1_::pre-pro-3xHA-EGFP-KDEL) for visualization of *AtCEP1* promoter activity. We found the specific up-regulation of *AtCEP1* in direct neighborhood of spreading leaf lesions thus likely representing cells undergoing PCD. Furthermore, we found a strong resistance of *atcpr5* mutant plants against infection with *E*. *cruciferarum*. Loss of *AtCEP1* had no obvious influence on the strong resistance of *atcpr5-2* mutant plants against infection with *E*. *cruciferarum*. However, the area of necrotic leaf lesions associated with *E*. *cruciferarum* colonies was significantly larger in *atcpr5-2* as compared to *atcep1 atcpr5-2* double mutant plants. The presence of AtCEP1 thus contributes to AtCPR5-controlled PCD at the sites of powdery mildew infection.

## Introduction

Programmed cell death (PCD) is a genetically determined, highly regulated process in all multicellular organisms and a prerequisite for successful development. PCD eliminates tissues and cells serving temporary functions during development such as tapetum cells in anthers and suspensor cells connecting the embryo to the mother plant or nucellus cells of a mature ovule [1–4]. Plants furthermore limit the spread of fungal or bacterial pathogens under execution of PCD at the site of infection in a mechanism called the hypersensitive response (HR) [5].

Diverse classes of proteases are involved in PCD, including cysteine proteases, serine proteases, aspartic proteases and metalloproteases [6,7]. A unique group of papain-type cysteine endopeptidases (CysEPs) is specific for plant PCD and characterized by a C-terminal KDEL endoplasmic reticulum (ER) retention signal (KDEL CysEPs) with RcCysEP from castor bean (*Ricinus communis*) as the founding member [8–10]. KDEL CysEPs are not present in mammals or fungi, but are ubiquitous in plants [11]. KDEL CysEPs are synthesized as pre-pro-enzymes and are co-translationally transferred into the ER, where the pre-sequence signal peptide is removed. KDEL CysEPs can be stored as enzymatically inactive pro-enzymes in ER-derived compartments [10,12–14]; upon acidification, the KDEL CysEPs are released and the pro-sequence together with the C-terminal KDEL endoplasmtic reticulum retention signal are removed for activation of the enzyme [10,15] (as described in detail previously [13]). The mature, enzymatically active KDEL CysEPs exhibit unusual broad substrate specificity. KDEL CysEPs are unique in being able to digest not only cytoplasmic components in tissues that collapse during final stages of PCD; they furthermore are able to digest extensins that form the basic scaffold for cell wall formation (as described in detail previously [16]). The broad substrate specificity is due to the active site cleft of the KDEL CysEPs that accepts a wide variety of amino acids including proline and glycosylated hydroxyproline of the hydroxyproline rich glycoproteins of the cell wall [17]. The respective amino acids, which are decisive for this generally more open appearance of the active site cleft, together with the amino acids defining the catalytic pocket are highly conserved among all known KDEL CysEPs [11].

In *Arabidopsis*, three KDEL CysEPs—CEP1 (At5g50260), CEP2 (At3g48340), and CEP3 (At3g48350)—have been identified that are expressed in tissues undergoing developmental PCD (as described in detail previously [13,16]). Furthermore, *CEP1* was found to be expressed in late response to biotic stress stimuli in the leaf (as described in detail previously [18]). Two *CEP1* T-DNA insertion lines (SAIL_158_B06 and SALK_01306, both carrying the T-DNA insertion within the 3^rd^ exon) showed enhanced susceptibility to powdery mildew caused by the biotrophic ascomycete *Erysiphe cruciferarum*. A translational fusion protein of CEP1 with a three-fold hemaglutinin-tag and the green fluorescent protein under control of the endogenous CEP1 promoter (P_CEP1_::pre-pro-3xHA-EGFP-AtCEP1-KDEL) rescued the pathogenesis phenotype demonstrating the function of CEP1 in restriction of powdery mildew disease. *atcep1* knockout plants transformed with the non-functional reporter including EGFP without the mature CEP1 subunit (P_CEP1_::pre-pro-3xHA-EGFP-KDEL) retained susceptibility to *E*. *cruciferarum*. The spatiotemporal *CEP1*-reporter expression during fungal infection together with microscopic inspection of the interaction phenotype suggested a function of CEP1 in controlling late stages of the compatible interaction including late epidermal cell death (as described in detail previously [18]).

Defense responses in *Arabidopsis* are regulated by multiple signal transduction pathways in which salicylic acid (SA), jasmonic acid (JA), and ethylene (ET) function as key signaling molecules. Mutants such as *cpr5* constitutively activate these defense pathways [19]. The *CPR5* gene (*CONSTITUTIVE EXPRESSION OF PR GENES 5*, At5g64930) is a regulator of expression of pathogenesis related genes and participates in signal transduction pathways involved in plant defense and development such as leaf senescence or flowering. It codes for nuclear envelope membrane protein [20]. Loss of *CPR5* leads to spontaneous expression of chlorotic lesions and reduced trichome development [21,22]. The *cpr5* plants were found to be constitutively resistant to virulent pathogens such as the bacterial pathogen *Pseudomonas syringae* and the oomycete *Hyaloperonospora arabidopsidis* [19,21]. We found in public expression data that *CEP1* (At5g50260, Affymetrix ATH1 probe set ID 248545_at; GEO accession GSE5745; this is unpublished work) is constitutively up-regulated in *cpr5* mutants [www.genevestigator.com; 23]. We used the *cpr5-2* mutant allele that has a point mutation in the fourth exon leading to a premature stop codon (Trp477stop) [24, 25] in order to analyze a possible contribution of the *CEP1* upregulation to chlorotic leaf lesions in *cpr5*. We measured an increase in *CEP1* expression in *cpr5-2* mutant, which coincided with the appearance of leaf lesions. The expression of *CEP1* was particularly evidenced in leaf cells that surround the chlorotic lesions and presumably underwent cell death. Furthermore, we found a strong resistance of *cpr5-2* against infection with *E*. *cruciferarum* and studied the pathogenesis and cell death phenotypes in *cep1 cpr5-2* double mutants as compared to the single mutants. This suggests a contribution of CEP1 to CPR5-controlled cell death.

## Materials and methods

### *Arabidopsis* mutant and reporter plants

We used homozygous knockout mutants for *cep1* (SAIL_158_B06; T-DNA insertion within the third exon) [18]. For imaging the functional proenzyme of CEP1 by confocal laser scanning microscopy (CLSM), we used the functional reporter P_CEP1_::pre-pro-3xHA-EGFP-AtCEP1-KDEL that rescued the *cep1* knockout phenotype when expressed in *cep1* [18].

A homozygous *cpr5-2* mutant allele (NASC stock code N3770) with a point mutation in the fourth exon leading to a stop codon (Tpr477stop) [25] was obtained and confirmed by sequencing: a 653 bp fragment comprising half of the fourth exon including the stop codon and six bp of the 3’UTR was amplified by PCR using the primers cpr5-2 fw GGCTCCTCGTAAGTGTCTTCAGC and cpr5-2 rv GGTCTGACTATGCTTGAGACGAG. The resulting PCR product was sequenced using the primers cpr5-2 seq fw CGATCATCAGGTACGAAGC and cpr5-2 seq rv GTCACGTTTATAGGACCG. Homozygous *cep1 cpr5-2* double mutant plants were obtained by crossing. In order to monitor *CEP1* promotor activity in cells of the *cpr5-2* mutant background, we made homozygous double mutants by crossing *cpr5* mutants and *cep1* knockout mutants harboring the non-functional reporter P_CEP1_::pre-pro-3xHA-EGFP-KDEL that exhibits a translational fusion protein of the necessary CEP1 targeting sequences together with a three-fold hemaglutinin-tag and the green fluorescent protein EGFP but lacking the mature CEP1 subunit under control of the endogenous *CEP1* promoter [18].

### Leaf infection with powders mildew and symptoms rating

Inoculation and assessment of disease progression was carried out as described [18].

### Necrotic leaf area measurement using fluorescence microscopy

Callose depositions in *Arabidopsis* cells were visualized by methyl blue (Sigma Aldrich Chemie GmbH, München, Germany) staining. For this purpose, leaves of *cpr5-2* single mutant plants and of *cep1 cpr5-2* double mutant plants, respectively, were harvested 5 days post inoculation (dpi) with *E*. *cruciferarum* and were discoloured in ethanol-acetic acid glacial (EtOH-HAc; 6:1). The discoloured leaves were rinsed with distilled water and transferred into 67 mM K_2_HPO_4_ buffer for 10 minutes followed by incubation for 2 h in the staining solution (0.05% methyl blue in 67 mM K_2_HPO_4_) in the dark and by direct analysis using fluorescence microscopy (Olympus BX61TRF, Japan).

Only areas of necrotic leaf lesions associated with powdery mildew colonies were measured using their fluorescence intensity. Areas as measured by fluorescence intensity under excitation by 340 nm wave length thus represented necrotic cells, but also other callose-enriched structures such as encasements of established haustoria. Sixty five fluorescing areas detected on 19 leaves on *cpr5-2* single mutant plants and on *cep1 cpr5-2* double mutant plants, respectively, were photographed and the fluorescing areas were quantified using ImageJ graphical analysis software version 1.48v [26]. To this end, the colour threshold was set to hide noise signals of unspecific adsorbed staining. Regions of specific fluorescence signals were selected and their areas were measured. Subsequently the data were corrected for the callose depositions belonging to powdery mildew-induced papillae and encasements of established haustoria. For this purpose, the average areas of ten powdery mildew colonies that were not associated to necrotic leaf lesions, on five leaves of *cpr5-2* single mutant plants and of *cep1 cpr5-2* double mutant plants, respectively, were measured using their fluorescence intensity. These values were subtracted from the fluorescent areas of necrotic leaf lesions associated with powdery mildew colonies on *cpr5-2* single mutant plants and on *cep1 cpr5-2* double mutant plants, respectively, thus obtaining the area of a chlorotic leaf lesion associated with a powdery mildew colony.

### qRT-PCR

Primers used for qRT-PCR are: ACT8 qRT fw: TGAGACCTTTAATTCTCCAGCTATG; ACT8 qRT rv: CCAGAGTCCAACACAATACCG; CEP1 qRT fw: TCAGCCTGTTTCTGTTGCTATT; CEP1 qRT rv: CATCTCCCGGTAAACACTCC; CEP2 qRT fw: GCTGTTGCAAACCAACCTG; CEP2 qRT rv: TTCCACAAGATCCCGTAAACA; CEP3 qRTfw: GCTCACCAGCCTGTCTCTGT; CEP3 qRT rv: CGCATTCTCCGATAAACACA. qRT-PCR was carried out as described [18].

### Confocal laser scanning microscopy

Staining of callose and imaging of the non-functional (P_CEP1_::pre-pro-3xHA-EGFP-KDEL) or functional (P_CEP1_::pre-pro-3xHA-EGFP-AtCEP1-KDEL) reporter plants was carried out as described [18].

## Results

### The proenzyme of CEP1 is associated with the haustorial complex encased in callose

**Fig 1:**
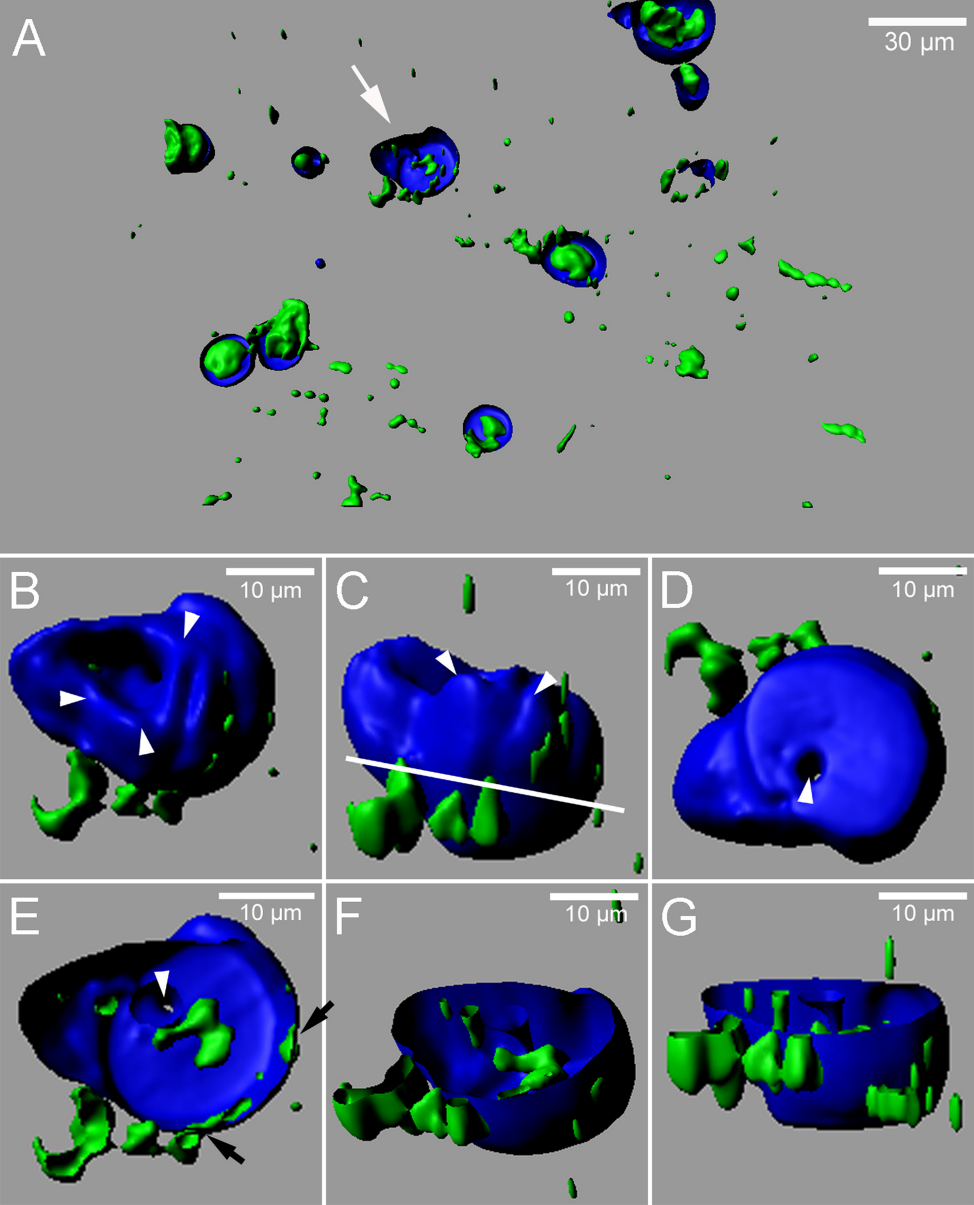
Three-dimensional reconstruction of fluorescence signals from the functional proenzyme of CEP1 (green) and callose depositions (blue) in epidermal cells of *Arabidopsis* invaded by haustoria of *E*.*cruciferarum*. (A) Overview about several interaction sites with callose-encased haustorial complexes in the epidermis of Arabidopsis leaves at 11 dpi. CEP1 proenzyme is visible in epidermal cells and in and around encased haustoria that are shown in an optical section and reconstructed in 3 dimensions for lower z-sections. (B-G) Accumulation of the CEP1 proenzyme at the side and within a haustorium that is encased with callose. The same haustorium is also highlighted with an arrow in A. (B) View from the cell surface. At this site, little CEP1-loaded ER is visible at the periclinal cell cortex (arrowheads) (C) View from the side of the haustorium. Arrowheads indicate the rim of the encasement closest to the periclinal cell cortex (D) Bottom up view. Arrowhead indicates incomplete closure of the callose encasement (E-G) Callose-encased haustorium cut at the line indicated in panel C viewed from different angels. Arrowhead indicates incomplete closure of the callose encasement. Black arrows indicate sites where CEP1 signals are visible at both the cytosolic and the haustorial side of the encasement. Original pictures were recorded by CLSM and reconstructed into a 3-demensional picture by the IMARIS software (Bitplane).

We previously described that cells attacked by *Erysiphe cruciferarum* express the proenzyme of CEP1 from the functional reporter P_CEP1_::pre-pro-3xHA-EGFP-AtCEP1-KDEL in the endoplasmic reticulum (ER) and accumulate it around haustoria. This led to the speculation that CEP1 could limit haustorium functions, which could explain enhanced growth of *E*. *cruciferarum* on *cep1* mutant plants [18]. To get a more detailed impression of whether CEP1 could indeed closely associate with haustoria, we combined live cell imaging of P_CEP1_::pre-pro-3xHA-EGFP-AtCEP1-KDEL reporter plants with imaging of callose after staining with the aniline dye methyl blue at 11 dpi. Three dimensional reconstructions of fluorescence signals recorded by CLSM suggested that the proenzyme of CEP1 is closely associated with callose-encased haustoria and can be even found within encasements of haustorial complexes (Fig 1A). Closer inspection of fluorescence signals suggested that the CEP1-containing ER is present on both sides of the callose encasement, the cytoplasmic side and the haustorial side (Fig 1B–1G). The ER seemed to either span the encasement or the ER that was associated with the haustorium was enclosed during development of the encasement. In some haustorial encasements, GFP signals were strong but we were unable to further resolve the position of the GFP signal within or around the haustorial complex.

### *CEP1* is specifically upregulated in *cpr5-2* mutants in leaf cells undergoing cell death that surround the chlorotic lesions

**Fig 2:**
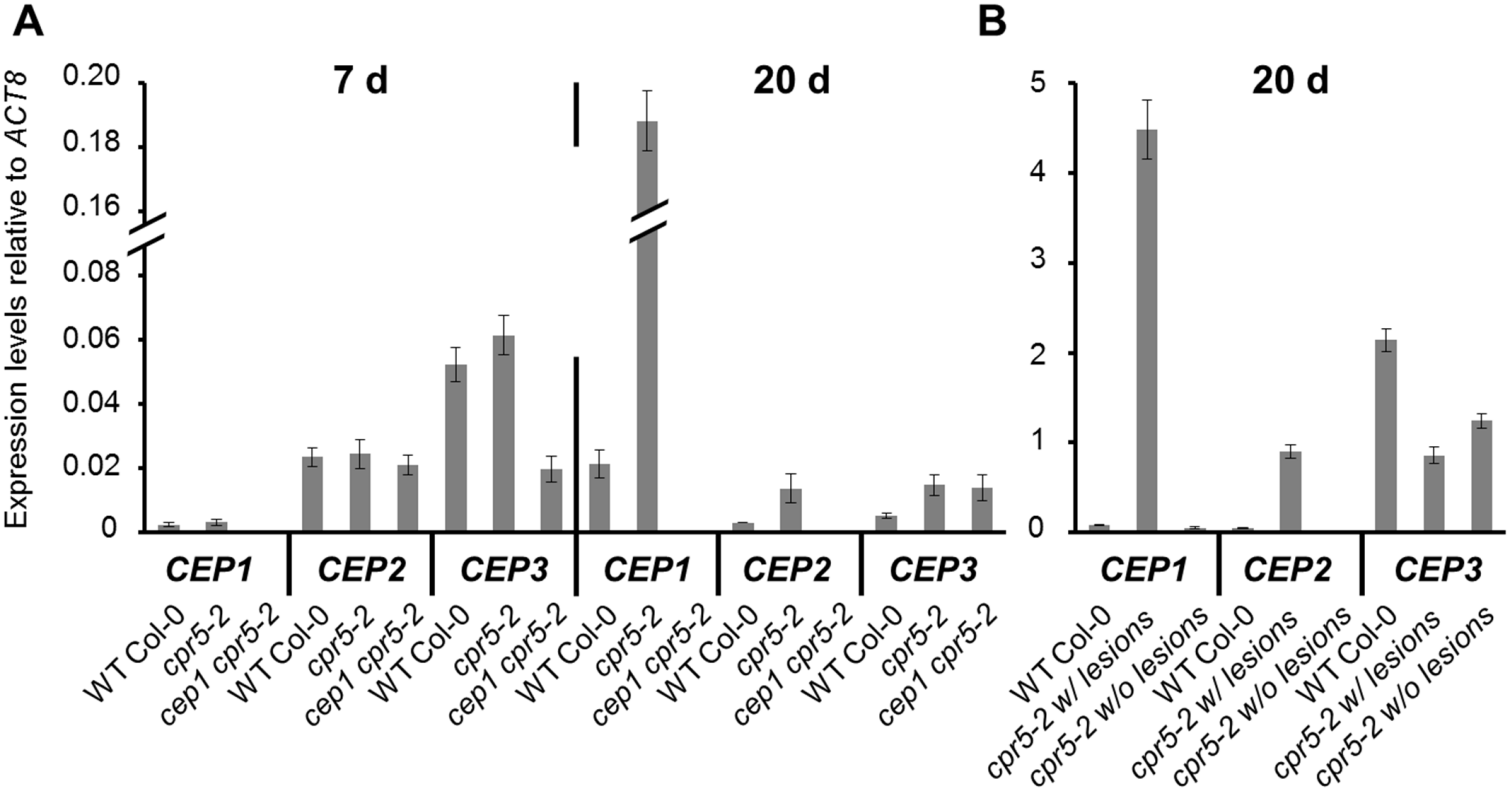
*CEP1* is specifically upregulated in *cpr5* mutants in leaf cells undergoing cell death that surround the chlorotic lesions. Relative gene expression of *CEP1*, *CEP2* and *CEP3* was measured in 7 and 20 days old seedlings of wild type Col-0 as well as *cpr5-2* single and *cep1 cpr5-2* double mutant plants. Four leaves with visible lesions from four different plants of each genotype, respectively, were collected in one tube for isolation of RNA (A). Eight—twelve leaf pieces with or without lesions from six different plants of each genotype, respectively, were collected in one tube for isolation of RNA (B). qRT-PCR was performed with *CEP1*, *CEP2* and *CEP3* gene-specific primers. Expression levels were relatively set to the reference gene *ACT8*. Error bars represent standard deviations due to three technical replicates. The results were similarly reproduced in a second independent experiment (biological replicate). Furthermore, the data for the *cep1 cpr5-2* double mutant plants were similarly reproduced with a second independent line. *cpr5-2* w/lesions, leaf pieces with lesions; *cpr5-2* w/o lesions, leaf pieces without lesions.

In publicly accessible global gene expression analysis of the *Arabidopsis atcpr5* mutant (GEO accession GSE5745) displayed in Genevestigator [23], we found that *CEP1* transcripts are significantly more abundant in two-week-old *cpr5-2* mutants than in wild type. CPR5 is considered as a negative control element of plant programmed cell death and effector-triggered immunity through cell cycle control [27]. We used the *cpr5-2* mutant allele that has a point mutation in the fourth exon leading to a stop codon (Trp477stop) [24,25]. Quantitative RT-PCR confirmed up-regulation of *CEP1* in the *cpr5-2* mutant allele correlating to the appearance of leaf lesions. *CEP1* was not expressed to higher values in leaves of seven day old *cpr5-2* seedlings before lesions were visible (Fig 2A left). An about ten-fold up-regulation of *CEP1* was found in leaf rosettes of 20 day old *cpr5-2* plants and correlated in time with the occurrence of spontaneous leaf lesions (Fig 2A right). On the other hand, *CEP2* and *CEP3* exhibited no strong expression in 20 day old rosette leaves in connection with the appearance of leaf lesion formation (Fig 2A). When we separated leaf rosettes of 20 day old *cpr5-2* mutants into leaves with and without visible necrotic lesions, only necrotic leaves showed about 50-fold up-regulation of *CEP1* transcripts (Fig 2B). Again, *CEP2* and *CEP3* did not show comparably high expression in leaves with necrotic lesions as compared to *CEP1*.

**Fig 3:**
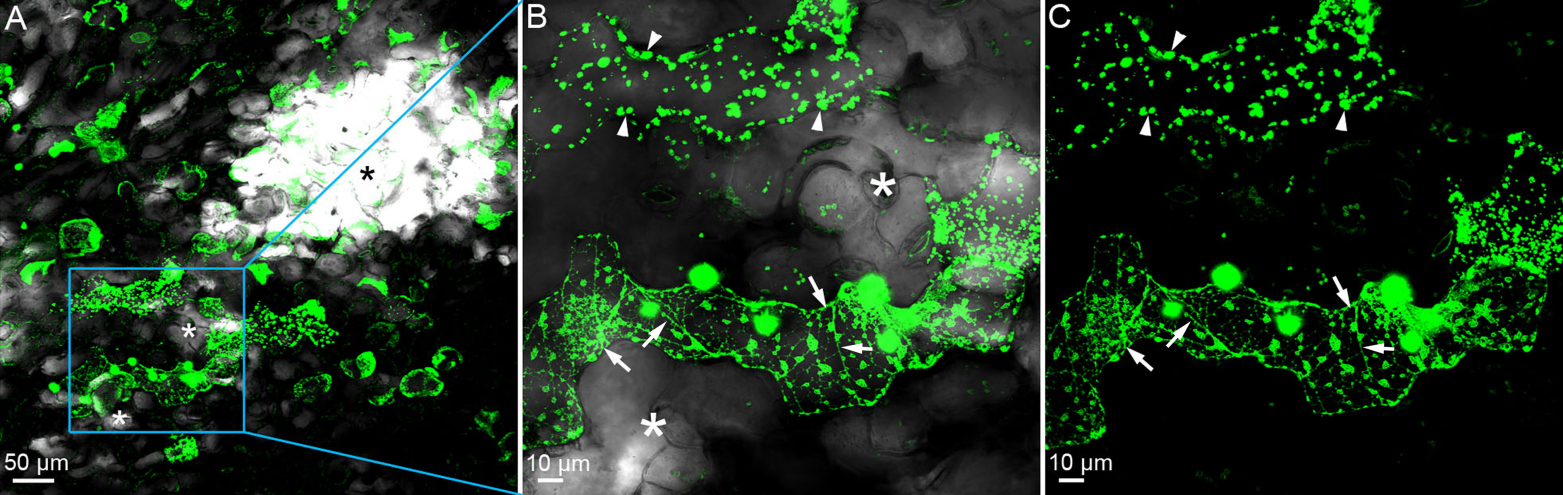
*CEP1* is specifically expressed in leaf cells undergoing cell death that surround the chlorotic lesions. **A**. The white area (black asterisk *) indicates a chlorotic lesion in the terminal stage; the blue box indicates the representative tissue, where the enlarged image in **B** and **C** can be found. **A and B**. The white asterisks (*) indicate cells in spreading chlorotic lesion presumably undergoing programmed cell death. **B**. Merge of bright field image and EGFP. **B and C**. The pattern of fluorescence shows partially a reticular pattern typical for the endoplasmic reticulum (arrows) and partially a punctate pattern suggesting endoplasmatic reticulum disintegration in cells that likely underwent PCD (arrow heads). **A, B** and **C**. EGFP indicates the expression of the non-functional reporter construct P_CEP1_::pre-pro-3xHA-EGFP-KDEL.

We made homozygous double mutants by crossing *cep1* knockout mutants harboring the non-functional reporter P_CEP1_::pre-pro-3xHA-EGFP-KDEL and *cpr5-2* mutants in order to visualize leaf cells that would express *CEP1* (Fig 3) and to study the impact of *cep1* knockout mutation on the *cpr5*-mediated resistance or cell death phenotype (see below). Strikingly, promoter activity of *CEP1* was exclusively confined to cells in direct neighborhood of chlorotic lesions (Fig 3A, asterix). Leaf cells in areas more distant to the chlorotic lesion exhibited only autofluorescence but no specific GFP signal as identified by wavelength scanning with the confocal laser scanning microscope. The pattern of pre-pro-3xHA-EGFP-KDEL fluorescence showed partially a reticular pattern typical for the ER and partially a punctate pattern of disintegrated ER in cells that likely underwent PCD during spreading of lesions (Fig 3B and 3C).

### *cpr5-2* mutants are powdery mildew resistant

**Fig 4:**
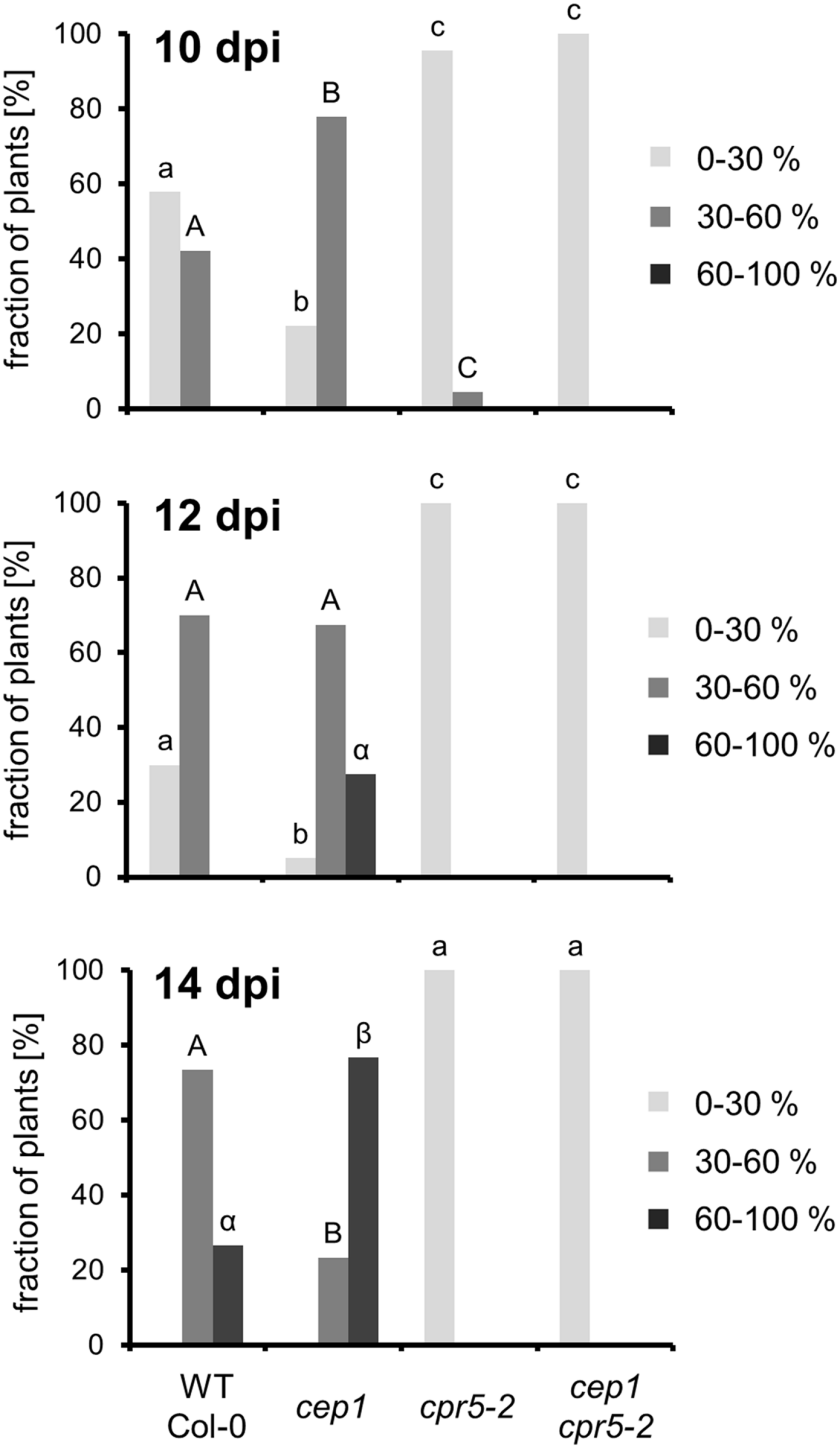
*cpr5-2* mutants are powdery mildew resistant. Disease symptoms of wild type, *cep1* and *cpr5-2* single mutants and *cep1 cpr5-2* double mutant plants upon leaf infection with *E*. *cruciferarum* were scored after visual inspection of the whole plant 10, 12 and 14 days post inoculation (dpi). Distribution of infected leaves in the three categories was carried out as described [18]. Infected leafs were distributed in the three categories <30%, 30–60%, and >60% diseased leaf area [18]. Columns of the same colour marked with different letters indicate statistically different groups (*p* < 0.05), and columns marked with identical letters indicate groups not statistically different (*p* > 0.05) according to the ANOVA- and Duncan test, and represent the frequency of plants distributed in the three categories of susceptibility. Data represent the respective means of eight experiments from independent inoculation events of the mutants with the corresponding parent background control. Each experiment comprised five plants per line.

**Fig 5:**
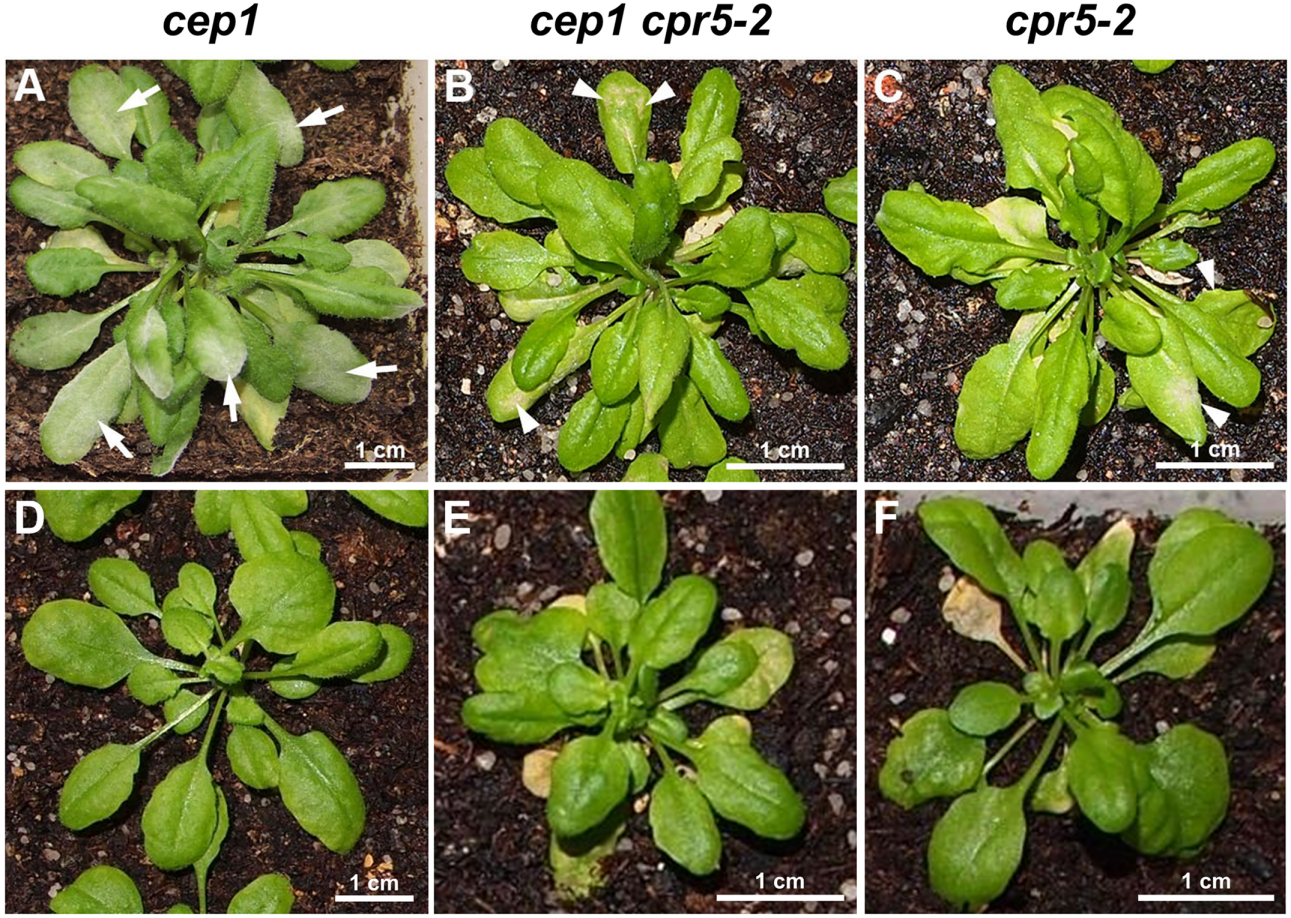
Powdery mildew disease phenotypes caused by the biotrophic ascomycete *Erysiphe cruciferarum*. (A) *cep1* knockout mutants developed the usual symptoms upon infection with powdery mildew (arrows). (B and C) In contrast, the *cep1 cpr5-2* double mutant plants and the *cpr5* single mutant plants are resistant to *E*. *cruciferarum*; necrotic lesions are indicated by white arrow heads; please note the scaling bar: *cpr5* and *cep1 cpr5-2* mutants are dwarf. Plants in (A-C) are shown 11 days post low density inoculation. (D-F) Non-infected plants at the time of inoculation.

We observed a strong resistance of *cpr5-2* mutant plants against infection with *E*. *cruciferarum* (Figs 4 and 5). *Arabidopsis* susceptibility to *E*. *cruciferarum* was scored by visual examination of the whole plant 10, 12 and 14 day after inoculation (Fig 4). The wild type Col-0 control plants developed the usual symptoms within 10 and 14 day after inoculation by exhibiting in time an increasing leaf area covered by powdery mildew symptoms with approximately 75% infected leaves in the category of 30–60% symptomatic leaf area and 25% in the category of >60% symptomatic leaf area14 day after inoculation. The *cep1* knockout plants again exhibited more severe symptoms as compared to wild type with approximately 25% infected leaves in the category of 30–60% symptomatic leaf area and 75% in the category of >60% symptomatic leaf area 14 days after inoculation. In marked contrast to these susceptible genotypes, the *cpr5-2* mutant plants showed exclusively leaves with powdery mildew symptoms in the category of <30% symptomatic leaf area throughout the investigation time (Figs 4 and 5).

### CEP1 contributes to powdery mildew induced leaf lesions on *cpr5-2*

We analyzed the homozygous *cep1 cpr5-2* double mutant obtained by crossing the *cep1* knockout mutants harboring the non-functional reporter P_CEP1_::pre-pro-3xHA-EGFP-KDEL and the *cpr5-2* mutants, in order to study the impact of *cep1* knockout mutation on the *cpr5*-mediated resistance or cell death phenotype as compared to the single mutants. Interestingly, loss of *CEP1* had no obvious influence on the strong resistance of *cpr5-2* mutant plants against infection with *E*. *cruciferarum* as analyzed by macroscopic symptoms rating (Figs 4 and 5). All leaves of the *cep1 cpr5-2* double mutant fell into the category of <30% symptomatic leaf area throughout the investigation time. The *cpr5-2* mutant phenotype appeared thus epistatic over the *cep1* mutant phenotype (Figs 4 and 5).

**Fig 6:**
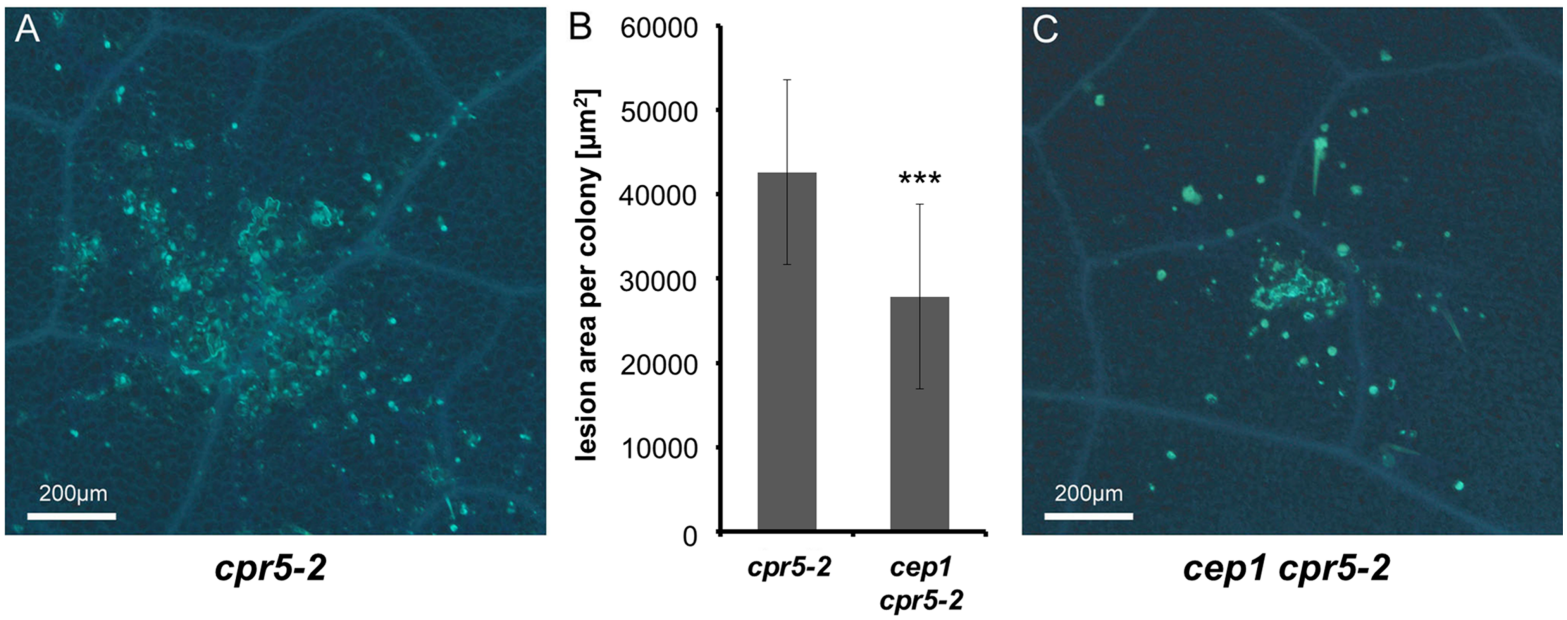
CEP1 contributes to powdery mildew induced leaf lesions on *cpr5-2*. **(A)** Representative leaf lesion on *cpr5-2* single mutant plants. **(B)** Comparison of the necrotic leaf lesion area per colony on *cpr5-2* single and on *cep1 cpr5-2* double mutant plants. Columns show the area of a necrotic leaf lesion per *E*. *cruciferarum* colony (five spores/mm^2^) five days post inoculation (dpi) measured by fluorescence intensity. The areas showing fluorescence above a certain threshold intensity were measured. Data present the mean of 65 lesions on 19 leaves on *cpr5* mutant plants and on *cep1 cpr5-2* mutant plants, respectively. Error bars are standard deviations. Differences between *cpr5* single and *cep1 cpr5-2* double mutants are highly significant after two sided Student’s *t*-test (*p* < 0.001, ***). **(C)** Representative leaf lesion on *cep1 cpr5-2* double mutant plants.

Our results suggested a strong activity of the *CEP1* promoter in spatiotemporal association with lesions developing in *cpr5-2* mutant plants (Fig 3). We therefore undertook a microscopic analysis of *cpr5-2* single mutant plants in comparison to *cep1 cpr5-2* double mutant plants at 5 days post inoculation with *E*. *cruciferarum*, when the powdery mildew growth still allows for the observation of single fungal colonies often associated with necrotic leaf lesions. Callose depositions were visualized by methyl blue staining in *Arabidopsis* cells at papillae, encasements of established haustoria as well as whole cells that die in the course of leaf lesion formation or epidermal hypersensitive response (Fig 6). The area of each necrotic leaf lesion associated with an *E*. *cruciferarum* colony was measured by fluorescence intensity. These average necrotic leaf areas were significantly larger in *cpr5-2* single mutant plants (Fig 6A and 6B) as compared to *cep1 cpr5-2* double mutant plants (Fig 6B and 6C). The presence of CEP1 thus contributed to the powdery mildew induced PCD and supported necrotic lesion formation.

## Discussion

### CEP1 is associated with encased haustorial complexes

Callose encasements of haustorial complexes is considered as a basal defense reaction of Arabidopsis against powdery mildew fungi, which cannot fully suppress this response [28]. Similarly, we interpreted spatiotemporal association of *CEP1* expression with late pathogenesis of *E*. *cruciferarum* as a contribution to basal resistance that limits fungal colonization success even in compatible interaction [18]. Plant ER has been observed before in close spatial proximity of developing and mature haustorial complexes [28–32]. Here, we visualized the presumable proenzyme ER-resident CEP1 around and within haustorial encasements at 11 days post inoculation. We further speculate that CEP1 could be released from a leaky ER or be actively secreted during late development or senescence of haustoria and come into contact with the haustorium. Direct activity of the protease on the haustorial complex or function in late epidermal cell death (as described in detail previously [18]) possibly explains the function of CEP1 in restricting late fungal development.

### *CEP1* is de-regulated in association with *CPR5*-controlled PCD

Publicly available gene expression data drew our attention to *CPR5* as a possible regulator of *CEP1* expression. Our targeted expression analyses of *CEP1* in young asymptomatic and older leaves showing spontaneous leaf lesion development supported that *CEP1* is overexpressed during de-regulated PCD in *cep5* mutants. With the findings that CPR5 controls cell cycle and PCD during development and effector triggered immunity to leaf pathogens of *Arabidopsis* [24,27] and the observation that CEP1 contributes to pathogen-induced epidermal cell death, it appeared possible that CEP1 supports an CPR5-controlled cell death program. Indeed, similar to CEP1, CPR5 controlled *E*. *cruciferarum*-induced leaf cell death albeit in the opposite direction. This was associated with near-complete powdery mildew resistance of *cpr5-2* mutants, which appears, based on previously published and our data, generally resistant to many hemibiotrophic and biotrophic pathogens [21,27,33,34].

### CEP1 contributes to CPR5-controlled PCD at sites of powdery mildew infection

Double loss of function mutants of *CPR5* and *CEP1* retained powdery mildew resistance and spontaneous lesion phenotype observed in the *cpr5-2* single mutant. Hence, the *cpr5-2* mutant phenotype was largely epistatic over that of the *cep1* phenotype in terms of symptom formation of powdery mildew. However, microscopic inspections of the size of lesions that were associated with powdery mildew microcolony formation showed that CEP1 indeed contributed to lesion formation in *cpr5-2* mutants challenged by *E*. *cruciferarum*. Because these lesions have never been observed to a comparable extent in Col-0 before when challenged by *E*. *cruciferarum* and extended into the mesophyll tissue of *atcpr5* mutants, we conclude that CPR5 negatively controls a powdery mildew triggered form of PCD. This type of PCD is simultaneously supported by CEP1, such that the *cep1 cpr5-2* double mutant showed limited spreading of pathogen triggered cell death before leaf lesions became visible by naked eye. Together this supports that CEP1 acts in pathogen-triggered and CPR5-controlled leaf cell death although it does not represent a central switch in that type of PCD because visible lesions still appeared to an extent not readily distinguishable from that of the *atcpr5* single mutant. This study was carried out in an accession of *Arabidopsis* susceptible to powdery mildew. Since CPR5 acts in effector triggered immunity [24,27], it would be interesting to see if CEP1 also contributes to the hypersensitive response that is mediated by endogenous or ectopically expressed powdery mildew resistance genes [35,36] in *Arabidopsis*. Spatial association of *CEP1* expression with powdery mildew infection sites or *CPR5* controlled cell death reactions might be interesting in context of other genes that function in spatial association with cell death. For instance, the salicylic acid receptors NPR3 and NPR4 support or restrict cell death via the abundance of NPR1 in a manner that depends on salicylic acid gradients in spatial distance to the actual site of lesion formation [37]. It might be thus interesting to study CEP functions in further mutants that show alteration of spontaneous or pathogen-triggered cell death.

## Conclusion

Papain-type CysEPs have diverse functions in plant defense to pathogens [38]. Our data further suggest that the papain-type KDEL CysEP CEP1 fulfills its function in plant defense during late development of *E*. *cruciferarum* in close spatial association with the fungal haustorium and haustorial callose encasements. Additionally, CPR5 controls resistance to powdery mildew and PCD in response to infection by *E*. *cruciferarum*. In *cpr5* mutants, *CEP1* is overexpressed in spatiotemporal association with spontaneous cell death. CEP1 contributes to CPR5-controlled PCD triggered by *E*. *cruciferarum* but is not required to express high level powdery mildew resistance of *cpr5* mutants.
